# Adherence to Allergen Subcutaneous Immunotherapy is Increased by a Shortened Build-Up Phase: A Retrospective Study

**DOI:** 10.1155/2020/7328469

**Published:** 2020-02-18

**Authors:** Cristiano Caruso, Barbara Bramé, Diego Bagnasco, Alessia Cocconcelli, Valeria Ortolani, Valerio Pravettoni, Sergio Scarpa, Giuliana Zisa, Giovanni Passalacqua, Stefania Colantuono

**Affiliations:** ^1^Allergy Unit, Fondazione Policlinico A. Gemelli, IRCCS, Rome, Italy; ^2^Clinical Allergy & Immunology Unit, Legnano Hospital, Legnano, Italy; ^3^Allergy and Respiratory Diseases, IRCCS Policlinico San Martino, University of Genoa, Genoa, Italy; ^4^Allergy Outpatient Clinic, AUSL Reggio Emilia, Reggio Emilia, Italy; ^5^Allergy and Clinical Immunology Department, ASST Fatebenefratelli Sacco, Milan, Italy; ^6^Clinical Allergy and Immunology Unit, Foundation IRCCS Ca' Granda, Ospedale Maggiore Policlinico, Milan, Italy; ^7^Clinical Allergy and Immunology Unit, AUSL Parma, Parma, Italy; ^8^Allergology and Immunology Unit, Novara Hospital, Novara, Italy

## Abstract

*Introduction*. The poor long-term adherence is known to affect the efficacy of allergen immunotherapy (AIT). In the case of injection AIT (SCIT), one of the main determinants is the inconvenience for patients to undergo prolonged build-up phases. Thus, simplifying the time schedule of the induction protocol could be effective in increasing the adherence to SCIT.

**Methods:**

We backtracked the SCIT renewal orders, thanks to the cooperation of the manufacturing company, and we compared the long-term adherence of 152 patients, who were prescribed with an abbreviated build-up schedule (4 injections, allergoid) with that of 302 patients treated with the same product, but with the traditional build-up protocol (7 injections).

**Results:**

According to the patient-named refills, those patients on the abbreviated build-up were significantly more compliant at the 2nd and 3rd year of treatment compared to the other group (*p*=0.0001). The drop-out rate after one year was also significantly lower between the two groups (*p*=0.0001). The drop-out rate after one year was also significantly lower between the two groups (*p*=0.0001). The drop-out rate after one year was also significantly lower between the two groups (

**Conclusions:**

Abbreviating the build-up phase by reducing the number of injections significantly improves patients' adherence to SCIT.

## 1. Introduction

Like all the long-term treatments [1], allergen immunotherapy (AIT) is affected by poor patient compliance [2–4]. Only a small proportion of patients completes the third year of treatment, which is considered the minimum optimal treatment duration [5, 6]. While some studies suggest that the adherence to subcutaneous AIT (SCIT) is better than AIT via sublingual route (SLIT) [7, 8], it was documented that also the SCIT adherence is far from optimal [1]. One of the main reasons for the poor SCIT adherence is the inconvenience for commuting to receive the allergy injections, especially in the build-up phase [3, 4, 9, 10]. Therefore, it has been suggested that shortening the treatment schedules by reducing the number of injections could indirectly improve the adherence rate [11]. The present study was carried out to verify whether patients undergoing an abbreviated SCIT schedule were actually more compliant than patients treated with a classic SCIT scheme.

## 2. Methods and Patients

**Figure 1:**
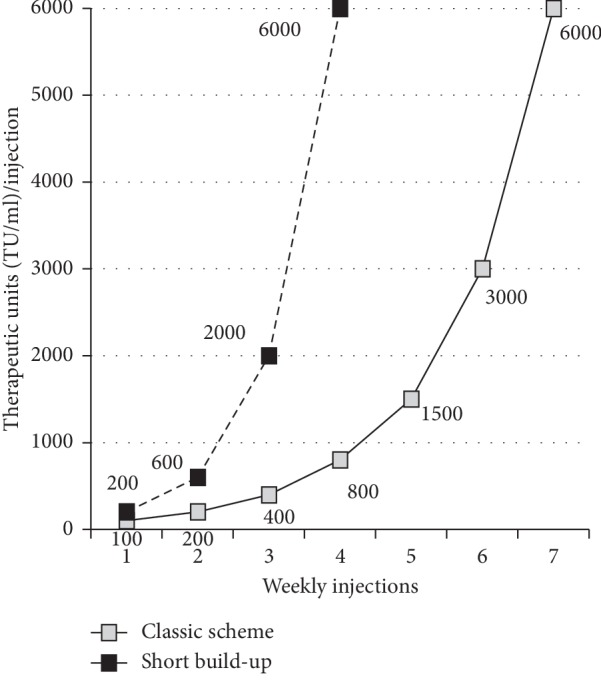
Comparison of classic and short build-up.

An abbreviated build-up scheme with pollen allergoids (Allergovit®, Allergopharma GmbH and Co. KG, Reinbek, Germany) has already proved to be safe and feasible [12]. This abbreviated protocol reaches the maximum dose with 4 injections, rather than the classic 7-injections scheme (Figure 1). To explore the possible impact of this schedule on patients' adherence, we retrospectively analyzed sales data of the manufacturing company to backtrack how many prescriptions each patient admitted to the abbreviated 4 injections preseasonal schedule has received; we compared these data with the adherence of patients treated with the same allergoid product, but with the standard 7 injections preseasonal schedule. The retrospective survey included 152 patients receiving the abbreviated build-up protocol (83 males and 69 females; mean age: 36.3 (min 7, max 65)), treated with different pollen extracts starting at least 4 years before. The comparison group was made of 302 consecutive patients treated with the same product in the same period but with the classic scheme, with comparable age and gender (171 males and 131 females; mean age: 35.9). We considered only patients who started the treatment at least 3 years before to allow a sufficient observation period. Since reimbursement issues are deemed important for patients' adherence [10, 13, 14], we also compared the adherence of 59 patients coming from 3 centers with full reimbursement with that of 86 patients from 3 centers with no reimbursement at all. While “adherence” to SCIT is not a standard defined concept, we evaluated the number of patients treated for at least two years, the number treated for at least three years, the number treated for more than three years, and the number of drop outs after one treatment year only. Data were retrospectively collected from seven Italian centers. No ethical committee approval was required since the treatment is part of the standard of care. Any statistical conspicuity was validated with Fisher's exact test to determine statistical significance defined as *P* < 0.05.

## 3. Results

**Figure 2:**
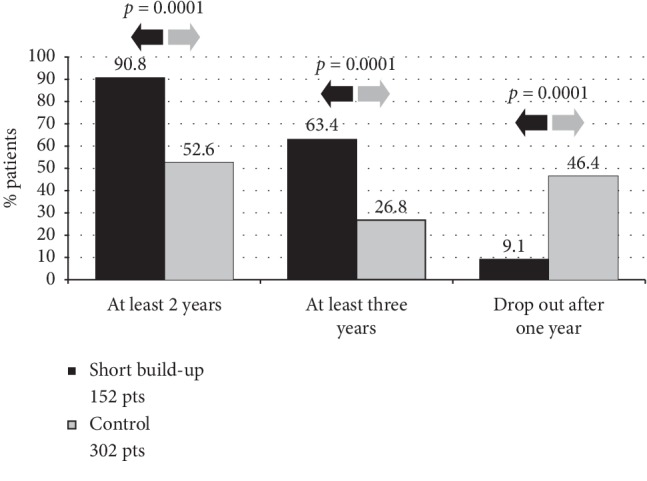
Adherence comparison between the two groups.

**Figure 3:**
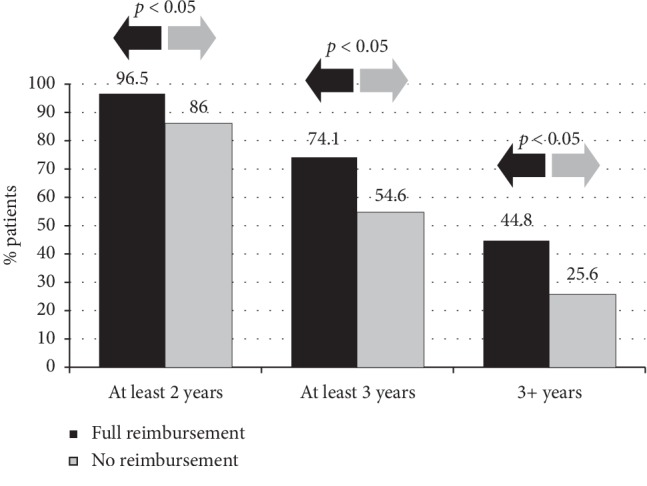
Impact of reimbursement on adherence.

90.8% of patients treated with the abbreviated schedule completed at least 2 treatment years, while 63.4% continued for at least 3 years. By comparison, in the control group, the adherence to ≥2 treatment years was only 52.6% (*p*=0.0001, Fisher exact test) and to ≥3 treatment years it decreased to 26.8% (*p*=0.0001) (Figure 2). Furthermore, the drop-out rate in the control group only after one treatment year was 46.4%, while in the abbreviated group, it was 9.1% (*p*=0.0001) (Figure 2). Remarkably, 49 patients (32.2%) maintained the treatment for more than 3 years, with an average treatment duration of 4.6 years. As expected, reimbursed patients were found to be significantly more compliant at any given point (*p* < 0.05) compared with patients with no reimbursement (Figure 3).

## 4. Discussion

**Figure 4:**
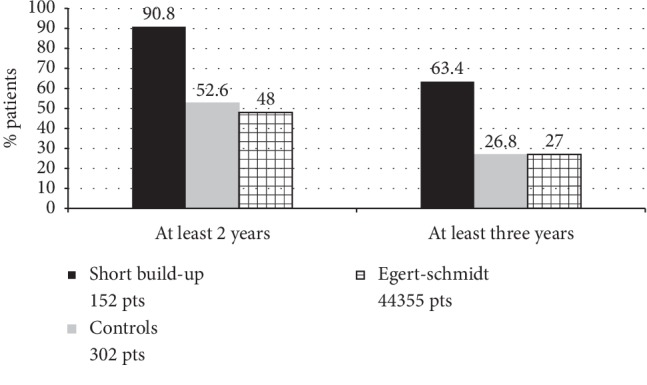
Adherence comparison with previous publication, using the same extract and the same schedule (16).

This retrospective survey confirms that shortening the build-up protocol of SCIT can significantly increase the SCIT adherence. This keeps with previous reports that the inconvenience for moving to the doctor's office to receive the injections is one of the main causes for poor SCIT compliance [3, 4, 9, 10]. Shifting from 7 to 4 preseasonal injections means to half the build-up phase, from 6- to 3-week span. Adherence to AIT is usually reported as the percentage of patients who maintain the treatment over a defined period of time. Some adherence studies report data for short periods of observation (6–12 months) [13–15], but guidelines recommend that in order to be effective, AIT must be continued for at least three years [5, 6]; therefore, we deemed important to investigate the three-year treatment persistence. In our survey, 63.4% of patients completed the requested third year of treatment. When considering persistence of treatment for 3 years, figures from published studies range from a low 16% to a high 89% [3, 8, 16–20]. But studies of AIT adherence are difficult to compare, due to the lack of a generally accepted adherence definition [4], different patients, methods of measurement [21], treatment schedules, practices organization [22], and reimbursement/costs issues [10, 19, 23]. Nevertheless, our data (63.4%) are comparable with those of Manzotti et al. study that found a 3-year adherence of 73.3% with a different pollen product, but with the same preseasonal 4 injections build-up schedule [18]. This study was carried out in Northern Italy, therefore, with the same type of patients and in a region with full AIT reimbursement. The 73.3% 3-year adherence in this study is absolutely comparable with the 74.1% we observed in our reimbursed patients, a confirmation that treatment costs are a major determinant of patients' compliance [10, 19, 23]. In this study, patients were allowed to choose the route of administration (SCIT or SLIT), and personal preferences already have proved to increase patients' adherence [15]. The most reliable comparison can be made with the publication of Egert-Schmidt et al. [16]. In this study, carried out by the manufacturing company, the authors, using the same method of backtracking AIT refills, evaluated the adherence of the same pollen allergoid product, administered preseasonally with the classic 7-injections scheme, in a very large survey (44,355 patients). The 3-year adherence in this group was 27%; absolutely, comparing with the 26.8% adherence, we found in our control group treated with the same extract and the same scheme (Figure 4). This further supports the validity of our observation. Of course, accelerating the build-up phase may involve some safety problems. Rush schedules can also increase AIT adherence [13], but it is well known that these schedules carry a higher risk of adverse reactions [24, 25]. The abbreviated updosing evaluated in this survey has instead already proved to be safe and well tolerated in previous studies, due to the hypoallergenic properties of allergoids [12, 26]. Backtracking the prescription refills is an objective, easy method to evaluate AIT adherence, but of course, it cannot guarantee that patients actually took the treatment [21]. Nevertheless, this observation is valid both for the short build-up patients and the control group, and still the difference between the two groups is highly significant. Furthermore, we made a second check in two centers, totaling 54 of our 152 patients, and all the refill data corresponded to the treatment courses actually carried out. In conclusion, abbreviating the build-up phase of an allergoid extract significantly increases the percentage of patients completing a 3-year course of SCIT. This, in turn, will improve clinical benefits and, at the same time, will reduce the waste of healthcare resources.

## Data Availability

The data used to support the findings of this study are available from the corresponding author (Cristiano Caruso) upon request.

## Abbreviations

AIT: Allergen-specific immunotherapy
SCIT: Subcutaneous immunotherapy
SLIT: Sublingual immunotherapy.
